# The Opportunities and Challenges Associated with the Implementation of Fourth Industrial Revolution Technologies to Manage Health and Safety

**DOI:** 10.3390/ijerph19020846

**Published:** 2022-01-13

**Authors:** Reneiloe Malomane, Innocent Musonda, Chioma Sylvia Okoro

**Affiliations:** 1 Department of Construction and Quantity Surveying, Faculty of Engineering and the Built Environment, University of Johannesburg, Johannesburg 2028, South Africa; imusonda@uj.ac.za; 2 Department of Finance and Investment Management, College of Business and Economics, University of Johannesburg, Johannesburg 2006, South Africa; chiomao@uj.ac.za

**Keywords:** innovations 4.0, innovation, 4iR, fourth industrial revolution, health and safety, challenges, opportunities, technologies, strategies, awareness

## Abstract

The fourth industrial revolution (4iR) technologies offer an opportunity for the construction industry to improve health and safety (H&S) compliance. Therefore, implementing the technologies is of top priority to improve the endless H&S incidents in construction projects, which lead to poor quality of work, late project delivery, and increased labour injury claims. Central to improving the nature of work and other industrial processes, the 4iR technologies have emerged. Concurrent with this trend is the importance of 4iR technologies in enhancing health and safety performance on construction sites. However, the implementation of 4iR technologies in the construction industry is faced with various challenges. Therefore, this paper reports on a study aimed at examining the challenges associated with implementing 4iR technologies in the construction sector in South Africa towards effective management of H&S. The study followed a systematic literature review, data collection using a questionnaire survey and thereafter, descriptive, and inferential analyses were conducted. The findings revealed that the implementation of 4iR technologies is challenged by a lack of adequate relevant skills, the unavailability of training capacities, expensive technologies, and negative perceptions such as fear of job loss by industry professionals. The findings are essential for the advancement of H&S research and implementation. In addition, the findings are important to industry decision-makers in order to elevate their awareness and promote the use of 4iR technologies to manage construction activities. The study implications include the need for the construction industry to collaborate with higher education institutions to conduct research and include 4iR in the curriculum.

## 1. Introduction

Health and safety (H&S) refer to the wellbeing and safety of humans from hazards. It includes programs, guidelines, and procedures that protect the safety, welfare, and health of any person engaged in work or employment, aiming to provide the ultimate safe working environment and reduce the risk of accidents and fatalities at work [1]. Furthermore, it aims at protecting the health of customers and the public, including anyone who might be affected by the worksite environment. The H&S within the construction industry in South Africa is governed by legislation [2]. The construction H&S legislation was introduced during the first world war, during a time when a high number of fatalities and injuries occurring [3]. The authors Ibem and Laryea [3] explain that the legislation derived from the 1918 Factories Act (Act no. 28 of 1918), which set the standard for the South Africa industry. The Act was later improved and developed to the 1941 Factories Machinery and Building Work Act (Act no. 22 of 1941) and others, namely the 1983 Machinery and Occupational Safety Act (Act no. 6 of 1983) which 10 years later became the 1993 Occupational H&S Act (Act no. 85 of 1993). Currently, the latest Acts are Amendment Act, No. 181 of the 1993 Labour Relations Act and Construction Regulations, 2003 [4]. This latest Act aims at ensuring the H&S of persons at work and persons who interact with construction plant and machinery at work. In addition, the Act is meant to protect persons from H&S risks arising from or in connection with the activities of persons at work and to set up an advisory council on occupational H&S [4]. Clearly, there has been a huge effort to improve health and safety in the construction industry through upgrades in legislation. However, [5] emphasizes that the construction industry is constantly experiencing poor H&S performance.

Ensuring the safety of employees and the general public is a huge challenge for the construction industry. The construction industry constantly fails to implement H&S measures in the workplace [1] and continues to battle with poor H&S implementation. The problem of poor H&S is global, and South Africa is also affected [6]. It is therefore crucial that H&S in the construction industry should receive more attention than ever before. Choi, Ahn and Seo [7] proposed the use of fourth industrial revolution technologies (4iR) in the industry to manage the H&S. The authors Choi, Ahn and Seo [7] reveal that the 4iR technologies at the initial stage of projects are capable of being used to plan for safety management and to detect possible risks and hazards, which may occur during the construction stage. Choi, Ahn and Seo [7] further indicated that these technologies have the capability of automating H&S management through real-time site monitoring of humans and vehicles movement while detecting hazardous zones on site which might result from ongoing activities and provide signals to humans when they are entering hazardous zones. This automated workplace monitoring uses a combination of 4iR technologies to enhance safety in work zones and of humans at the workplace [8].

Various studies have presented the benefits of using 4iR technologies in the construction industry. For instance, Nnaji and Karakhan [9] and Shamsudin, Mahmood, Rahim, Mohamad and Masrom [10] used the Virtual Reality (VR) tool to train staff at the worksites on H&S to avoid hazards. Likewise, Raphaelson [11] used drones in various construction projects to perform multiple tasks such as inspecting works, monitoring the safety of humans and movements of vehicles while detecting hazards. On the other hand, Ramage [12] presented real-time safety detection, alarms on possible dangers to humans and vehicles and report to centralized management systems through smart sensor technologies and management tools.

Additionally, the various studies have outlined the benefits and position of the industry on 4iR adoption in the construction industry. The study by Ikuabe, Aghimien, Aigbavboa and Oke [13] asserted that the level of awareness of digital technologies in construction is still low. As an example, Osunsanmi, Oke, and Aigbavboa [14] observed that although RFID can help in monitoring the safety of construction professionals, both the cost of procuring and low levels of technical ability have inhibited its adoption. Janse van Rensburg [15] revealed that there are only a few technologies used in the construction industry. Likewise, according to Alaloul, Liew, Zawawi and Kennedy [16] and Lau et al. [17], there are many 4iR technologies that can help enhance productivity and safety, however, the industry is far from implementing them. Osunsanmi, Aigbavboa, Oke and Liphadzi [18] opined that the level of willingness to adopt 4iR technologies is high, however, the level of possible integration is very low. In a study by Gaspar, Julião and Cruz [19], respondents were not sure of their ability to operate the 4iR technologies.

Despite these opportunities to enhance H&S management in the construction industry, the implementation of 4iR technologies is still very low [20]. When using the keywords “opportunities, challenges, 4iR technologies, construction, health, safety, South Africa” on Google Scholar, the search retained zero results. Likewise, when searching on Scopus using keywords such as “opportunities” AND “challenges” AND “4iR technologies” AND “construction” AND “health” AND “safety” AND “South Africa”, zero results was retained. In addition, when searching Web of Science using the keywords: “opportunities challenges 4iR technologies construction health safety South Africa” only one (1) journal article was retained. Therefore, there is need for an extensive investigation of opportunities and challenges associated with implementing the 4iR technologies in the construction industry to manage H&S in South Africa.

Therefore, this study aimed at examining the opportunities and challenges within the construction sector in South Africa towards 4iR technologies implementation. The study commenced by identifying technologies that are available for use or adoption, primarily driven by the 4iR. Secondly, the current opportunities available for implementing 4iR technologies in the South African construction industry, and thereafter the challenges associated with implementing 4iR technologies, were identified. Based on the identified opportunities and challenges, the study proposes strategies to implement 4iR technologies in the South African construction industry. The approach may be applicable to other developing nations with similar characteristics.

## 2. Materials and Methods

The study employed a multi-pronged approach. Firstly, a systematic literature review was conducted to scope, plan, identify, screen, and assess the current body of knowledge on the subject. Materials for the review of literature on the factors of H&S non-compliance, strategies, opportunities, and challenges in the construction industry for 4iR technologies were sourced from journals listed in the Web of Science, Scopus, and Google scholar databases, including the International Journal of Environmental Research and Public Health and Safety Science in view of their safety-focused research areas. The multiple search sources were used for a comprehensive search in order to identify relevant publications as adopted by Qi, Razkenari, Costin, Kibert and Fu [21] and Akram, Thaheem, Nasir, Ali and Khan [22]. The former [21] argued that collating existing research articles from various perspectives can help understand the state-of-art on an issue. The literature search strategy included identifying keywords. In the current study, the keywords were: Innovations 4.0, Innovation, 4iR, H&S, Challenges, Opportunities, Technologies, Strategies, Awareness and South Africa. The review focused on papers published in the last ten years (2010 to 2020). According to Manda and Dhaou [23], this period coincides with implementing South Africa’s digital transformation plan. The article searches in the various databases on the subject yielded 102 papers. The exclusion and inclusion criteria methods were used to select, screen, and identify the most suitable literature carefully. In addition, the articles were checked for duplication. After the qualification criteria and the duplicates were removed, 54 articles were retained and reviewed for the study.

Secondly, a questionnaire was used to collect data from respondents using a 5-point Likert scale as it was deemed suitable for this kind of study [24,25,26]. The questions presented in the questionnaire were derived from the literature review on the opportunities and challenges associated with the implementation of 4iR technologies to manage H&S in the South African construction industry [25]. Closed-ended questions were used to limit answers [27]. The questionnaire was validated through a face validity scientific test to ensure the intended questions were maintained [28]. The questionnaire was set up as follows: the first section, section A, explored the participants’ background information. The second section contained questions about the opportunities to manage H&S using 4iR technologies. The third section included the challenges associated with implementing 4iR technologies. The respondents were required to indicate the extent to which they agreed with statements regarding the challenges and opportunities (identified from the literature review) for implementing 4iR technologies on a 5-point scale where 1 = Strongly disagree (SD); 2 = Disagree (D); 3 = Neutral (N); 4 = Agree (A) and 5 = Strongly agree (SA).

The convenience sampling was used to easily reach the targeted sample of construction safety personnel, including Safety Officers, Foremen, Site Engineers, Site Agent, Construction Manager, and other professionals [18,29]. The respondents were selected on the basis that they manage H&S daily and are aware of 4iR in the industry. The safety personnel were sampled from construction sites that were running at the time of the study in the City of Johannesburg, Gauteng province. According to Habib’s [30] report from the University of Witwatersrand in Johannesburg, Gauteng is the first province to introduce 4iR technologies with major clients from Johannesburg. Thus, the study selected the City of Johannesburg.

A total of 110 respondents were targeted from the population on construction sites running at the time. This sample size was observed to be sufficient for studies of this nature to yield meaningful results, as was undertaken by Smallwood and Emuze [31], whereby 92 participants were included. Out of 110 distributed questionnaires, 88 were returned, which is an 80% return rate for the study. A return rate of less than 40% is unacceptable and yields validity issues, whereas a study with a return rate of 60% is considered suitable [32]. Therefore, with a return rate of 80%, the researchers deemed the data sufficient for analysis. The returned questionnaires were then screened and analysed.

Thirdly, the data collected was captured on an excel spreadsheet and later transferred to the Statistical Package for the Social Sciences (IBM SPSS Statistics) version 26 software for quantitative data analysis. Before analysing the results, missing values were checked and excluded [25]. Further, normal probability plots, histograms, and scatter plots were used to identify outliers and normality. The results showed that the data was not normally distributed, with few outliers emerging. The outliers were kept in the data because the respondents were the targeted population and represented valuable information [33].

This study employed both descriptive and inferential data analyses methods. The descriptive was used to determine the relative importance of the variables empirically using mean score (MS) and standard deviation (SD) values. The exploratory factor analysis was used to summarize the most significant variables and reduce them to quickly analyse and interpret the results and produce patterns and groupings that can be easily read and comprehended [34].

Preliminary analysis for the Exploratory Factor Analysis (EFA) included assessing the correlation matrix and testing with the Kaiser Meyer Olkin (KMO) and Bartlett’s Sphericity tests [25]. The data should be suitable for factor analysis, and to do so, the sample should be verified [25]. Smaller samples result in the items’ correlation coefficient being less reliable [35]. In addition, the suitability of the strength of the inter-correlation among the variables should be verified [36]. The factor analysis is questionable when no correlation is above 0.3 [25]. When Bartlett’s test of Sphericity is less than 0.05 and KMO have a minimum 0.6 index, the factor analysis is appropriate [37,38,39]. Once the factor assessment had been conducted, factor extraction followed in order to determine the factors that could be utilized to indicate the inter-relationships between the items; lastly, the factor rotation was conducted [25]. The Varimax type of rotation method was used to rotate and combine the items in groups, and relative importance was represented as orthogonal for ease of interpretation [36]. The Kaiser’s criterion and scree tests were used to choose factors to be retained, with an eigenvalue greater than one [34]. On the other hand, to interpret the EFA output, a cut-off value of 0.4 was used for the factor loadings [34]. This was conducted in order to ensure significant outcomes and to avoid non-significance issues [36].

## 3. Results

### 3.1. Results from Secondary Data

This section presents results from the systematic literature review on challenges on H&S regulations compliance, 4iR in construction, available 4iR technologies in the construction industry, opportunities, and challenges in the construction industry to manage H&S, implementing 4iR technologies and the strategies to adopt 4iR in the South African construction industry.

#### 3.1.1. H&S Regulations Compliance Challenges

The hazardous nature of the construction site often causes incidents and managing this issue has been a problem for years [40]. However, there is the latest Acts that governs safety management on site such as the Occupational H&S Amendment Act, No. 181 of 1993 Labour Relations Act and Construction Regulations, 2003 [4]. Some factors affect the performance of H&S in the construction industry.

Al-Bayati [41] discovered that the size of a contracting firm determines the level of compliance that occurs on construction projects; the smaller the company the poor the H&S performance. Windapo and Oladapo [42] disclosed that subcontractors, lower management and supervisors in small firms do not implement H&S regulations. Additionally, the small contracting firms have a high rate of poor H&S due to a lack of funds [43]. Consequently, the majority of fatalities on construction sites are caused by non-compliance with H&S regulations, which is not seen as an important factor mostly in small businesses [42].

Hon, Hinze and Chan [44] opined that the accidents and injuries encountered on-site are due to ignorance by both the employees and employers. Furthermore, non-compliance results from negligent attitude, poor knowledge, and lack of understanding of the legislation and the profit focus attitude [42]. The Department of Labour charges employers a huge amount for the penalties for non-compliance to enforce the implementation of the H&S regulations [45]. However, this does not yield any change. H&S challenges cannot be ignored as this will lead to non-improvement of poor safety performance. Schwab [46] revealed that the 4iR technologies could help solve the safety problems on site. Thus, this study proposes the implementation of 4iR technologies.

#### 3.1.2. The 4iR in Construction

The 4iR is South Africa’s hope in boosting the current declining economy [47]. More investment in the automation of construction industry is the one most important factor to boost the economy. However, the challenges of investing in the 4ir technologies are not covered in the paper. Through the reviewed literature, it was observed that there are benefits that this revolution brings to the construction industry. According to Choi, Ahn and Seo [7], the technologies will benefit the industry by resulting in the best form of accident prevention by protecting workers in hazardous areas through the provision of real-time data collection for safety reporting and incidents prevention. Furthermore, the technologies help provide greater visibility, better reporting, accountability, better communication, and improved workflows. These technologies will improve productivity, save on project time and cost, reduce workplace hazards, and push construction into the future by enhancing safety zones and mobility [8]. In addition, the authors emphasized that the technologies could shape and improve H&S in the construction industry. Other benefits of 4iR include fast transaction, reduced cost and easy usage [3].

Nevertheless, the construction companies are afraid of the political landscape, concerned about potential job losses and their impact on labour forces and infrastructure challenges and that smart technologies cannot be adopted in an unstable environment. According to Olojede, Agbola and Samuel [48], South African construction companies have long-standing resistance to change and rather focus on traditional methods, poor productivity, and tight competition in the industry. Therefore, they are afraid that digitisation might affect sustainability of the industry. The article covered the challenges of embracing the 4ir in the construction industry. The Government should play a major role in promoting technologies through policies, standards, and procurements to make it easy for small and medium enterprises to adopt the technologies [49].

#### 3.1.3. Available 4iR Technologies in the Construction Industry

The issue of poor H&S implementation cannot be ignored in the construction industry. To fight this, Lee, Shariatfar, Rashidi and Lee [50] opined that the implementation of 4iR technologies needs to be in place to enhance the management of H&S at the workplace. The 4iR comprises technologies that work concurrently from the pre-planning stage to real-time construction site management of works, humans, and machines [46]. The following technologies presented in Table 1 below were identified from the literature review, particularly in the construction industry.

Woodhead, Stephenson and Morrey [68] opined that the use of IOT and RFID together will monitor and control H&S on construction sites. Meanwhile, Nnaji and Karakhan [9] have in real life shown that a multiuser friendly tool (virtual reality) can train the workforce to safely erect and dismantle a tower crane. The tool operates in a virtual form, providing steps to set up and operate the tower crane. Likewise, Li, Yan and Liu [69] have practised drones’ operation in various construction projects. The drone is a vehicle used to perform various tasks, inspect works, monitor safety of employees, and identify hazards. Furthermore, tools such as smart sensors are identified to detect and report on any dangers at the workplace [69]. Getuli, Ventura, Capone and Ciribini [53] explained that BIM had been used as a semi-automatic tool to help in checking multiple safety regulations and safety plan and detect clashes to help safety performance.

The literature review has disclosed that these technologies are within 4iR in the construction industry. From the findings, it is discovered that technologies available in the construction industry include artificial intelligence, robotics, the internet of things, 3D printing, drones, building information modelling, smart devices, virtual reality, geographic information system (GIS), 4D computer-aided designs, radio frequency identification (RFID), ultra-wide band (UWB), global navigation satellite system (GNSS), global positing system (GPS) and sensors. The technologies such as IOT, RFID, VR, sensors, drones, and BIM can be adopted to manage H&S by the management team, train relevant stakeholders on safety measures and monitor the safety of workers on site.

#### 3.1.4. Current Opportunities to Manage H&S Using 4iR Technologies

The management of H&S entails proper training, communication, monitoring and controlling. These are made easy by 4iR technologies, which encourage safety training using virtual reality, augmented reality, inspection through automation, simulation training, and collaborative (human-robot) teams [9]. Furthermore, technologies such as BIM can enhance controlling and monitoring the overall project from the design phase to the closeout phase [52].

Choi, Ahn and Seo [7] corroborated that the technologies benefit from fending off accidents, generating greater visibility, easing reporting procedures and accountability, providing healthier communication, and ameliorating workflow. On the other hand, Zhang, Cao, and Zhao [70] asserted that technologies such as GPS and RFID help monitor workplace operations, transfer communication, detect harmful areas, and report on possible incoming dangers. Nnaji, Gambatese, Lee and Zhang [8] found that the use of tools such as speed reduction systems (SRS) decreases and monitor the speed of vehicles, intrusion prevention and warning system (IPWS) warns workers and vehicles drivers when entering an intrusion zone and human-machine interaction detection system (HMIDS) warns the worker and driver of equipment collision.

From the literature findings, the opportunities existing in the construction industry to manage H&S include decreasing fatalities, more time to solve more complex tasks, greater visibility, better reporting, accountability, and communication, improved workflow, monitoring, control, and data collection. Current opportunities also include cost savings, reduced injuries, construction gains and sustainability, improved safety inspections, and better information management.

#### 3.1.5. Challenges of 4iR Technologies Implementation

The implementation of 4iR in the South African construction industry will attract investments [71]. However, implementation is faced with the challenges outlined below.

##### Construction Firms’ Level of Interest and Views

Implementing 4iR technologies in the construction industry is perceived to be too expensive to adopt and maintain rather than innovate [72]. On the other hand, Japheth and Kiprotich [2] disclosed that the professionals in the industry show no interest in implementing the technologies and resist change to their traditional ways. This low interest in embracng 4iR results from a lack of specialized professionals, technical skills and the client not insisting and strategizing on implementing the technologies. Likewise, Bayode, van der Poll and Ramphal [73] pointed out that the construction industry is faced with insufficient electricity, unavailability of financial resources, poor accessibility to wireless broadband and lack of skills as notable barriers. Moreover, construction firms choose to stick to the proven methods of performing works and perceive adopting new technologies as risky [74].

##### The Size of Projects and Availability of Resources

Most companies are not implementing the 4iR technologies because the projects they are involved in are small-to-medium in size. These companies are undecided about adopting the technological assets due to the cost affordability of implementing and maintenance [15]. On the other hand, Alade and Windapo [75] opined that the industry lacks dynamic capabilities for adopting technologies. Studies show that lack of education and unalignment of labour supply and demand are the challenges in the industry. The narrative that digitization will cause job losses is a significant worry of many companies, while another impediment is the lack of digital skills [76]. Gaspar, Julião and Cruz [19] asserted that the fear of job losses is a challenge to adopting 4iR. Furthermore, Kariem [77] opined that lack of technical capacity with the absence of policy and regulation on 4iR implementation is another barrier to adopting the technologies.

##### Unavailability of Funds

The South African Cooperative Governance and Traditional Affairs is keen to support the civil construction industry to move to digital operations; however, it is faced with challenges in moving to e-governance [78]. The challenges include developing policies for affordable access to developing mobile broadband infrastructure and adequate skills to develop e-government services. Furthermore, Alade and Windapo [75] disclosed that the most significant challenge to implementing the technologies include the high cost of obtaining innovation and the high cost of training.

The literature revealed that the challenges associated with the implementation of 4iR technologies include lack of innovation, cost of adoption, fear of change and job losses, inadequate training capabilities, lack of interest, unavailability of specialists, unskilled technical support, lack of client insistence, insufficient electricity, and unavailability of financial resources. Others include lack of access to the wireless broadband, preference to traditional methods, size of the project, lack of education, misalignment of labour supply, unavailability of funds from the client and lack of adequate skills.

#### 3.1.6. Implementation Strategies

The implementation of 4iR technologies is a critical factor in managing H&S in the construction industry. However, the implementation in South Africa is challenged with issues discussed in the above section. It is of great importance to mitigate these challenges. Osunsanmi, Aigbavboa and Oke [79] indicated a low level of awareness of these technologies and a low level of understanding of how the technologies operate to manage works in the industry. This level of awareness results from the lack of understanding of the benefits in the construction industry. This suggests that the adoption of 4iR can be improved through training, workshops, and seminars.

On the other hand, Aghimien, Aigbavboa, Aghimien, Thwala and Ndlovu [80] concludes that the industry has a low awareness rate of the benefits of 3D printing. Therefore, higher education institutions should improve the training on these technologies in their syllabuses [81]. In addition, case studies should be conducted, a module in 4iR should be incorporated in the construction department and professionals should be educated on the technologies [82]. Ignoring these strategies of overcoming the implementation challenges of these technologies will result in the industry lacking the required skills [77] and thus will face difficulties in the future.

The literature review revealed that the suggested strategies towards implementing 4iR technologies in the construction industry include educating relevant parties, technology enlightenments, campaigns, training programmes, skill developments, workshops, seminars, government enforcement, regulation of the technologies and H&S policies on technologies.

### 3.2. Empirical Findings

This section presents results on the opportunities and challenges of implementing 4iR technologies in the construction industry in South Africa. 

#### 3.2.1. Opportunities for 4iR Implementation in H&S Management

Descriptive and inferential analysis results on the 4iR technologies opportunities to manage H&S in the construction industry are presented in this section.

##### Descriptive Results on the Opportunities

Table 2 shows that better information management was the most important opportunity for implementing 4iR technologies. This variable was ranked first, with MS of 4.21 and SD of 1.00; improved workflow was ranked second (MS = 4.20; SD = 0.98); improved safety inspections followed (MS = 4.16; SD = 1.00); better accountability was ranked fourth (MS = 4.14; SD = 0.91); preventing accident was ranked fifth (MS = 4.13; SD = 0.97). On the other hand, the least important variables were more time to solve more difficult issues (MS = 3.98; SD = 0.93); construction gains sustainability (MS = 3.90; SD = 1.04) and cost savings (MS = 3.47; SD = 1.30). These suggest that some opportunities are perceived more important in managing H&S than others.

##### Inferential Analysis Results (Factor Analysis) on Existing Opportunities

An exploratory factor analysis (EFA) of the opportunities revealed the opportunities existing to manage H&S in the construction industry using 4iR technologies. Before conducting the PCA, the suitability of the data for factor analysis was evaluated. The inspection of the correlation matrix showed the presence of coefficient of over 0.30 for most of the variables which was suitable for factor analysis. The Kaiser-Meyer-Olkin (KMO) measure of sampling adequacy achieved a value of 0.82, which is greater than the recommended minimum value of 0.6 and Bartlett’s Test of Sphericity value was significant (0.000) (Table 3). Therefore, the factorability of the data was possible.

The findings revealed that three components could be retained (Table 4). The eigenvalues greater than one (1) Kaiser’s criterion was adopted. The 3 components had eigenvalues of 7.60, 1.44 and 1.05, and contributed 50.69, 9.57 and 7.01% of their variance respectively. The three groups have a total cumulative variance of 67.27% of the total importance, which indicate their significance from the fifteen opportunities selected. The factors from the principal component analysis with a loading of 0.4 should retain at least 3 components with a minimum of total variance extracted of 50% [83].

**Figure 1:**
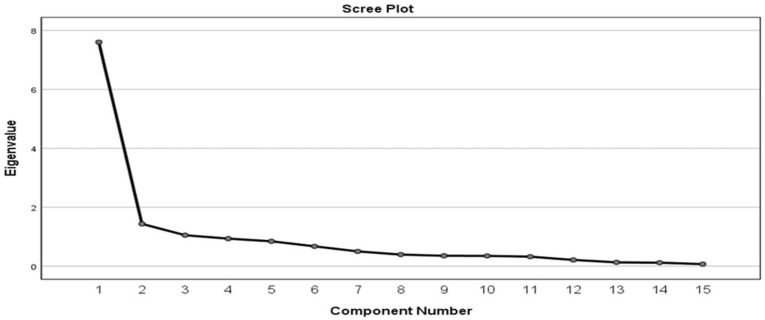
Scree plot for Opportunities.

Figure 1 shows the scree plot break after the third component. The steep slope represented the larger components, while the gradually decreasing components presented the rest of the variables with eigenvalue less than 1. The three groups located on the steep slope were retained. Varimax rotation was carried out to interpret the three groups of the opportunities existing in the construction industry for 4ir technologies to manage H&S. This gave rise to the pattern matrix presented in Table 5.

Findings from the factor analysis identified the following three components. The percentages represent component loadings, respectively.

Component 1 has eight items allotted to it, as seen in Table 5. These eight items belong to site specific benefits as concluded by Meno [36] of the opportunities existing in the construction industry for 4ir technologies to manage H&S. The component presents: construction gains sustainability (91.00%), decreases fatalities (79.30%), reduce injuries (70.50%), improve workflow (70.10%), better collection of data platform (68.50%), improved safety inspections (65.70%), better information management (63.20%) and better accountability (58.10%). The percentages represent components loadings, respectively.

Component 2 has four items allotted to it as seen in Table 5. These four items belong to company specific benefits as concluded by Meno [36] of the opportunities existing in the construction industry for 4ir technologies to manage H&S. The component presents: saves on cost (44.10%), create greater visibility (74.10%), more time to solve more difficult issues (66.10%) and preventing accident (56.20%). The percentages represent components loadings, respectively.

Component 3 has three items allotted to it as seen in Table 5. These three items belong to project specific benefits as concluded by Meno [36] of the opportunities existing in the construction industry for 4ir technologies to manage H&S. The component presents: better reporting (97.90%), better communication (84.50%) and improve in controlling and monitoring (54.00%). The percentages represent components loadings respectively.

#### 3.2.2. Challenges in Implementing 4iR Technologies

Descriptive and inferential analysis results on the challenges associated with implementing 4iR technologies in the construction industry are presented in this section.

##### Descriptive Results on the Implementation Challenges

Table 6 shows that the high cost of technologies was the most significant barrier. This aspect was ranked first, with a mean score (MS) of 3.95 and a standard deviation (SD) value of 1.20; fear of job losses was ranked second (MS = 3.92; SD = 1.15). A lack of adequate skills ranked third (MS = 3.72; SD = 1.18); the lack of cost to adopt ranked fourth (MS = 3.58; SD = 1.21); the unavailability of training capabilities was ranked fifth (MS = 3.57; SD = 1.34). On the other hand, the least important challenges were lack of access to the wireless broadband, which ranked fourteenth (MS = 3.16; SD = 1.27); lack of interest ranked fifteenth (MS = 3.07; SD = 1.30); insufficient electricity ranked sixteen (MS = 2.84; SD = 1.49). These suggest that some challenges may pose more threat to the implementation of 4iR than the others.

##### Inferential Analysis Results (Factor Analysis) on Implementation Challenges

An exploratory factor analysis (EFA) was conducted on the challenges associated with implementing 4iR technologies in the construction industry. All nineteen (19) variables were retained. Principal component analysis (PCA) was utilized to analyse the 15 variables using SPSS version 26 software.

Before carrying out the PCA, the suitability of the data for factor analysis was evaluated. The inspection of the correlation matrix showed the presence of a co-efficient of over 0.30 for most of the variables, which was suitable for factor analysis. The Kaiser-Meyer-Olkin (KMO) measure of sampling adequacy achieved a value of 0.80, more significant than the recommended minimum value of 0.6 and Bartlett’s Test of sphericity value of 0.000 was acceptable. Therefore, the factorability of the correlation matrix is significant as shown in Table 7.

The results in Table 8 revealed the challenges associated with implementing 4iR technologies in the construction industry with their respective eigenvalues. The eigenvalues greater than one Kaiser’s criterion was adopted. A total of 4 challenges were retained: 6.10, 1.97, 1.72 and 1.43, with 32.08, 10.39, 9.03 and 7.51% of their variance, respectively. This means that the first group accounted for 32.08% required for challenges, the second group accounted for 10.39%, the third group accounted for 9.03%, and the fourth group accounted for 7.51%. The 4 groups have a total cumulative variance of 59.02% of the total importance, which indicate their significance from the nineteen challenges selected. The factors from the principal component analysis with a loading of 0.4 should retain at least three components with a minimum of total variance extracted of 50% [83].

**Figure 2:**
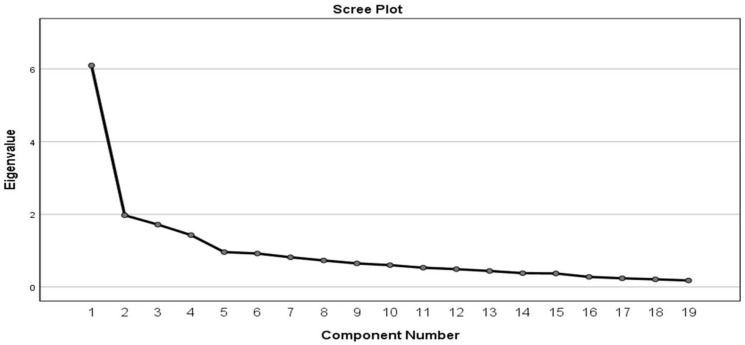
Scree plot for Challenges.

Figure 2 shows the scree plot break after the fourth component. The steep slope represents the larger components, while the gradually decreasing components represent the rest of the variables with eigenvalue less than 1. The four groups located on the steep slope were retained. This gave rise to the pattern matrix presented in Table 9.

Findings from the factor analysis identified the following four components. The percentages represent component loadings, respectively.

Component 1 has seven items allotted to it as seen in Table 9. These seven items belong to people-related factors that can affect 4iR implementation, as concluded by Odubiyi [84]. The component represents: unskilled technical support (83.20%), unavailability of specialist (82.90%), unavailability of training capabilities (65.00%), lack of adequate skills (62.40%), lacks client insistence (54.00%), lacks interest (64.40%) and prefer traditional method (40.30%).

Component 2 has three items allotted to it as seen in Table 9. These three items belong to management-inclined problems as concluded by Odubiyi [84]. The component represents: technologies are too expensive (74.60%), fear of change (64.40%) and lack of innovation (52.20%).

Component 3 has five items identified as cost-related problems [84]. The variables here include: insufficient electricity (45.20%), unavailability of financial resources (80.70%), unavailability of funds from client (70.30%), lack of cost to adopt (60.10%) and size of project (60.00%).

Component 4 has four items that belong to standardization problems associated with 4iR implementation [84]. The factors include: lack of education (79.50%), unalignment of labour supply (73.10%), fear of job losses (65.60%) and lack of access to the wireless broadband (61.20%). these relate to the enlightenment strategies.

## 4. Discussion

This section presents the findings of 4iR technologies opportunities that exist in the construction industry to manage H&S and challenges faced by the construction sector in implementing 4iR technologies. The findings from descriptive and factor analysis obtained in the previous results section are discussed in relation to the literature reviewed earlier in this research. The findings align with the results identified in literature. However, the scaling of respondents on the challenges differs.

### 4.1. Opportunities for 4iR Technologies to Manage H&S

The systematic literature review discovered the 4iR technologies opportunities that exist include decreasing fatalities, more time to solve more complex tasks, greater visibility, better reporting, accountability, and communication, improved workflow, monitoring, control, and data collection. Current opportunities also include cost savings, reduced injuries, construction gains and sustainability, improved safety inspections, and better information management.

The descriptive results ranked variables through MIS and standard deviation, respectively. The most significant variables were better information management, improved workflow, improved safety inspections, better accountability, and preventing accidents. Furthermore, the least essential variables include better communication, reduced injuries, more time to solve more complex issues, construction gains sustainability and saves on cost.

The factor analysis findings presented the groupings for these technologies, which are component one: site-specific benefits, including improved workflow, improved safety inspections, better information management, and better accountability as most important. Construction gains sustainability and reduces injuries were noted as the least important. Component two included company-specific benefits: preventing accidents as most essential and saves on cost and time to solve more complex issues as least important. Component three includes project-specific benefits, which include better communication as the least.

The benefits make H&S management easier and faster. Information flow and progress reporting are also easier to handle because these technologies are automated and can control themselves and suggest safer decisions. The recognition of all technologies available still needs to be raised because the opportunities of the technologies are not known by many safety personnel. Therefore, the strategies of implementation are mandatory for the management of H&S.

From the results, safety personnel perceived the most regarded opportunities for the implementation of 4iR were improved workflow, improved safety inspections, better Information management and better accountability. These are in line with the findings of Choi, Ahn, and Seo [7], who opined that the technologies will benefit in fend off accidents, generate greater visibility, ease reporting procedures and accountability and ameliorate workflow. This is probably because these technologies are automated and can control themselves and suggest safer decisions. On the other hand, Nnaji and Karakha [9] found that implementing these technologies is essential for enhancing construction sustainability using preventive tools that reduce injuries and accidents at the workplace. The authors’ finding contradicts the findings of this research, which perceived construction gains and sustainability and reduced injuries as less significant opportunities.

#### 4.1.1. Component Two: Company-Specific Benefits

The findings revealed that the most significant company-specific benefit was preventing accidents. The reason behind this perception might be the ability of these technologies to detect and prevent incidents. This matched with the results found by Nnaji and Karakhan [9], who suggested that the preventive tools make the site a controllable place that prevents accidents 

#### 4.1.2. Component Three: Project-Specific Benefits

The finding in this group described better communication as the least important of project opportunities in employing 4iR. Education on these technologies is lacking, which implies that safety personnel do not understand the operations of the automated workplace. The results mismatch with the findings of Choi, Ahn and Seo [7], who suggested that the technologies could provide healthier communication.

Extant literature showed that the use of 4iR technologies can improve safety of individuals and equipment at the worksite. Cases of injuries, accidents, and fatalities can be eliminated using sensors, RFID, GPS, and smart devices. Additionally, the implementation of the 4iR technologies will encourage safety training using Virtual Reality and or Augmented Reality and conducting inspections through automation. Further, 4iR can improve H&S practices/performance by simulating trainings. These technologies do not only benefit construction projects but also the organisation in planning and managing occupational H&S from the initial stage using tools such as BIM, IoT and 4D cad. These tools are able to produce safety management plans and be able to monitor the plan during process with the help of tools such as drones, GPS and GNSS. These tools are generally used for digital data collection from a targeted area and use it within BIM, IOT and 4D cad to compare with the planned H&S measures.

### 4.2. Challenges in Implementing 4iR Technologies

This section presents the challenges faced by the construction sector in implementing 4iR technologies. The findings from descriptive and factor analysis obtained in previous results section are discussed in relation to literature reviewed earlier in this research. The findings align with the findings identified in the literature that these exist. However, the scaling of respondents on the challenges differs.

The systematic literature review identified the challenges associated with the implementation, including too expensive technologies, fear of job losses, lack of adequate skills, lack of cost to adopt, and unavailability of training capabilities. On the other hand, the challenges with lesser risks include the unavailability of specialists, lack of education, lack of access to wireless broadband, lack of interest and insufficient electricity.

The descriptive results supported that expensive technologies, fear of job losses, lack of adequate skills, lack of cost to adopt and unavailability of training capabilities were significant challenges. Further, the least important challenges were unavailability of specialists, lack of education, lack of access to wireless broadband, lack of interest and insufficient electricity.

The factor analysis findings presented the groupings for these barriers, which are component one: people-related factors including lack of adequate skills and unavailability of training capabilities as the most important challenges and lack of interest and unavailability of specialist as the least important; and component two: management-incline problems which notes that costly technologies is the most critical challenge. Component three–cost-related problems included lack of cost to adopt as the major challenge and insufficient electricity as the least important factors; and component four: standardization problems which include fear of job losses as the most important variable and lack of education and access to wireless broadband as the least important.

The findings revealed that the unavailability of training capacities and lack of adequate skills are the main threats to implementing the 4iR technologies in the construction industry. The results are in line with Japheth and Kiprotich [2], who disclosed that the professionals are afraid to change the traditional way and adopt the new technologies and, most importantly, employ digital training. Likewise, Kariem [77] emphasized that poor technical capacity with no plan for improvement is another threat to adopting the new technologies. This is probably because 4iR technologies are perceived as conspiracies and not possible to implement in South Africa. Further, the findings are mirrored with the results of Moloi, Zibani, and Makhubela [78] who stated that developing e-government services in the industry is hindered by lack of adequate skills. The reason behind this might be that these tools are new in the country and there are no professionals or technicians acquainted with operating them.

The results also disclosed a lack of interest and the unavailability of specialists as the minor threats to implementing new technologies. However, this contrasts with the findings of Mahachi [72] who asserted that the construction industry lacks interest because of perceptions that it is impossible to implement these technologies. Further, the findings of Kariem [77] who concluded that the industry lack technical personnel to operate these technologies contrast this study’s findings.

The imagination and ignorance further hinder the implementation of these new technologies from perceptions. This is because the small and medium enterprises in the industry in South Africa mostly rely on government projects based on traditional operations. The results match with the findings of Mahachi [72] who asserted that the construction sector refuses to innovate to develop a strategic plan to eliminate this challenge but instead conclude that the technologies are too expensive.

The challenge of lack of cost to adopt this method was one of the greatest threats to the implementation of 4iR technologies. The truth is that implementing these technologies will not be easy because it might mean that most of the traditional ways would come to an end. This finding is mirrored with Janse van Rensburg [15] who concluded that small and medium projects with less management resources and asserts are not ready mostly financially to embrace the technologies. This is probably because projects of these sizes do not have enough funds to cover up the employment of the technologies. From the findings, the insufficient electricity challenge is less risk to the implementation of technologies. This mismatched with Bayode, Van der Poll and Ramphal [73] who emphasized that the industry is constantly experiencing insufficient electricity. This probably results from the country’s shortage of resource to generate adequate electricity to stakeholders.

Due to the level of unemployment is very high in South Africa, with many looking for jobs for a long time with no luck, the safety personnel of the construction industry see the adoption of technologies as the greatest threat to their jobs. This is perhaps why the fear of job loss was found to be one of the highest risks in implementing technologies in the industry, as was found in Gaspar, Julião and Cruz [19]. Likewise, Birkel, Veile, Müller, Hartmann, and Voigt [76] emphasized that job losses are a severe fear in the industry. Further, the challenges of lack of education and lack of access to wireless broadband were found to be less of a risk in adopting the technologies. However, when one does not have the knowledge or the skills to operate these tools, they will have difficulties working/operating the tools. Further, most technologies use wireless to operate and thus lack of education and access are serious barriers Birkel, Veile, Müller, Hartmann, and Voigt [76]. These findings are contrary to Bayode, Van der Poll and Ramphal [73], who asserted that access to wireless broadband is a severe challenge to the operation of these tools considering that the tools use much of wireless to operate.

The negative H&S issues prevalent in the construction industry need to be addressed. To mitigate the effects of H&S challenges, the use of 4iR technologies is key. The use of the technologies in turn can be enabled through raising the level of awareness achieved through providing training, conducting workshops and seminars. Further, to enhance skills and education on 4iR operations, institutions of higher education should incorporate 4iR practices in the syllabus. Additionally, professionals who are already working in the industry need to be provided education and skills either through professional training or short courses. Moreover, the paper recommends client incentives to drive 4iR technology adoption.

## 5. Conclusions

The construction industry is an essential sector contributing to the economic growth of South Africa. The industry is constantly experiencing poor H&S performance that leads to indirect H&S costs from injuries, fatalities and accidents claims. The government made efforts of establishing occupational H&S regulations and practices, however, the industry has found difficulties in practising the regulations. Therefore, this study proposes using 4iR technologies to manage H&S performance.

Implementing 4iR technologies will help reduce fatalities, injuries, and accidents, finally improving performance. Moreover, it will produce construction work that has fewer hazards. The implementation of 4iR technologies is faced with severe challenges. This study established the many issues faced by the construction industry in adopting 4iR technologies. Although the study presents findings from South Africa, the issue of 4iR adoption is faced by many developing countries. Hence, the study provides information to an international audience of Built Environment practitioners and scholars to compare the applicability of similar phenomenon and/or methodologies in their countries. The issues go beyond just low level of awareness as identified by several scholars. For example, construction companies do not implement these technologies because of the nature of contracts. Most companies rely on the national government for support and the size of their firms is also a factor. The companies rely on government contracts that have a standard approach for carrying out contracts. Therefore, there is a great need for the national governments to revisit their procedures, policies, and regulations to enable technologies to be implemented. Moreover, most companies lack the financial capability and are not sure if they can maintain the 4iR process; therefore, proper planning to accumulate financial resources should be established. The government and the construction firm should develop strategies to adopt and maintain these technologies in the industry to overcome the challenges faced by the industry to implement the technologies.

The issue of 4iR adoption is faced by many developing countries. Publishing this article in a global journal will ensure that the research is easily visible for broader audience, helping other scholars to compare the applicability of similar phenomenon and/or methodologies in other countries. Moreover, the subject of 4iR in health and safety management is still not fully comprehended on a global scale. Therefore, lessons even from local settings are useful at a global level.

## Figures and Tables

**Table 1:** The Available Technologies.

Technologies	Description/Function	Source
Radio Frequency Identification (RFID)	This comprises tags and readers system used to detect, alarm and transfer data using a wireless. This technology is used on humans and equipment on site to ensure that they are working in a safe environment	Osunsanmi, Oke and Aigbavboa [14]; Haupt, Akinlolu and Raliile [51]
Building Information Modelling (BIM)	Optimizes the integration of design, procurement, construction, end of use of building and structures. The technology is used during the pre-planning of site safety management to identify possible hazards which may arise during construction, and suggest solutions. During construction, BIM works as a semi-automatic technology that helps check multiple safety regulation and safety plan, detecting any clashes to ensure good safety performance is managed.	Smallwood, Emuze and Allen [52]; Getuli, Ventura, Capone, and Ciribini [53]
Artificial Intelligence (AI)	A technology that can replace humans, operating through computer techniques monitoring and controlling the safety of the workers, equipment, and the structure. The technology works faster than humans, and monitors and manages site H&S with urgency.	Niu et al., [54]; Gheisari and Esmaeili [55]
Third Dimensional (3D) Printing	A technology that automates the building of structures while creating a less hazardous environment.	Alhumayani, Gomaa, Soebarto and Jabi [56]
Robotics	Automates construction works, creates less hazardous zones and greater visibility of the site and performs tasks that are even difficult to humans. Robotics help in mitigating human injuries and decreasing accidents on-site.	Llale, Setati, Mavunda, Ndlovu, Root and Wembe [57]; Aghimien, Aigbavboa, Oke and Thwala [58]
Sensors	Analyzes measurements of health monitoring through centralized real-time information reporting. Sensing reduces construction safety fatalities by alarming workers of risks within their zones.	Hanus and Harris [59]; Zhou, Whyte and Sacks [60]
Ultra-Wide Band (UWB)	Uses three or more receivers positioned at the area to be monitored to detect anything that could cause safety issues at the workplace.	Jiang, Skibniewski, Yuan, Sun and Lu [61]
The Internet of Things (IOT)	Helps with the H&S management processes from the initial stage and during construction by providing automated H&S training and monitoring of humans and site vehicles and plants.	Tang, Shelden, Eastman, Pishdad-Bozorgi and Gao [62]
Smart Devices	Are tools attached on humans and plants, detecting possible hazards, monitoring their movements, computing the data and sounding alarms when nearing dangerous zones or objects.	Niu et al., [54]
Geographical Information System (GIS)	Collects the geographic distribution of onsite works using spatial relations, creating a protocol that results in ease of H&S management.	Fenais, Ariaratnam, Ayer and Smilovsky [63]
Drones	Captures through video big data from lower ground level to the highest heights of a construction site providing real-time movement of the works, detecting possible safety risks and providing feedback via audio communication. Further, this technology is used for the inspection of safety and wellbeing of humans and plants at the workplace.	Gheisari and Esmaeili [55]; Tatum and Liu [64]; Howard, Murashov and Branche [65]
Virtual Reality (VR)	Is applied as an automated H&S training providing visualization of real-time detection of hazards, and enhancing knowledge on safety management.	Zhou, Whyte and Sacks [60]
Four-Dimensional Computer Aided Design (4D CAD)	The information about the project activities is inserted in this technology. The information is then analysed, detect any possible risks and generate a safety management plan at the design stage.	Zhou, Whyte and Sacks [60]; Zhang, Sulankivi, Kiviniemi, Romo, Eastman Teizer [66]
Global Navigation Satellite System (GNSS)	Provides real-time monitoring of data of a large population from geosynchronous satellites, ensuring easy control and management of workplace safety.	Fenais, Ariaratnam, Ayer and Smilovsky [63]
Global positing system (GPS)	A positioning tool that uses wireless to track works and detect collision. It works as a security safeguard machinery in a robotic construction.	Li, Cheng and Chen [67]

**Table 2:** Existing Opportunities.

The 4ir Technologies Opportunities Existing in the Construction Industry to Manage H&S	$\bar{x}$	σX	R
Better Information management	4.21	1.00	1
Improved workflow	4.20	0.98	2
Improved safety inspections	4.16	1.00	3
Better accountability	4.14	0.91	4
Preventing accident	4.13	0.97	5
Better reporting	4.10	1.05	6
Improve in controlling and monitoring	4.09	1.02	7
Create greater visibility	4.09	0.85	7
Better collection of data platform	4.09	1.07	7
Decreasing fatalities	4.08	1.11	8
Better communication	4.06	1.09	9
Reduce injuries	4.02	1.03	10
More time to solve more difficult issues	3.98	0.93	11
Construction gains sustainability	3.90	1.04	12
Saves on cost	3.47	1.30	13

$\bar{x}$ = Mean item score; σX = Standard deviation; R = Rank.

**Table 3:** Measures of Sampling Adequacy.

Measures of Sampling Adequacy		
Kaiser-Meyer-Olkin		0.82
Bartlett’s Test of Sphericity	Approx. Chi-Square	879,900
	df	105
	Sig.	0.000

**Table 4:** Total Variance Explained.

Component	Initial Eigenvalues			Extraction Sums of Squared Loadings		
	Total	% of Variance	Cumulative %	Total	% of Variance	Cumulative %
1	**7.60**	50.69	32.082	6.096	**50.69**	50.69
2	**1.47**	9.57	42.471	1.974	**9.57**	60.26
3	**1.05**	7.01	51.504	1.716	**7.01**	**67.27**
5	0.935	6.23	64.069			
6	0.845	5.64	68.913			

Extraction Method: Principal Component Analysis. Values in bold represent the four components retained and their variance.

**Table 5:** Pattern Matrix.

	Component		
	1	2	3
Construction gains sustainability	0.91		
Decreases fatalities	0.793		
Reduce injuries	0.705		
Improve workflow	0.701		
Better collection of data platform	0.685		
Improved safety inspections	0,.657		
Better Information management	0.632		
Better accountability	0.581		
Saves on cost		0.441	
Create greater visibility		0.741	
More time to solve more difficult issues		0.661	
Preventing accident		0.562	
Better reporting			0.979
Better communication			0.845
Improve in controlling and monitoring			0.540

**Table 6:** Implementing Challenges.

Challenges Associated with the Implementation of 4iR Technologies	$\bar{x}$	σX	R
Technologies are too expensive	3.95	1.203	1
Fear of job losses	3.92	1.147	2
Lack of adequate skills	3.72	1.184	3
Lack of cost to adopt	3.58	1.210	4
Unavailability of training capabilities	3.57	1.335	5
Prefer traditional method	3.56	1.303	6
Unavailability of funds from client	3.55	1.372	7
Fear of change	3.55	1.330	7
Unavailability of financial resources	3.49	1.330	8
Unskilled technical support	3.49	1.268	8
Lack of innovation	3.44	1.353	9
Lacks client insistence	3.43	1.258	10
Unalignment of labour supply	3.38	1.187	11
Size of project	3.35	1.269	12
Unavailability of specialist	3.35	1.269	12
Lack of education	3.26	1.280	13
Lack of access to the wireless broadband	3.16	1.268	14
Lacks interest	3.07	1.302	15
Insufficient electricity	2.84	1.485	16

$\bar{x}$ = Mean item score; σX = Standard deviation; R = Rank.

**Table 7:** Measures of Sampling Adequacy.

Measures of Sampling Adequacy		
Kaiser-Meyer-Olkin		0.80
Bartlett’s Test of Sphericity	Approx. Chi-Square	649,148
	df	171
	Sig.	0.000

**Table 8:** Total Variance Explained.

Component	Initial Eigenvalues			Extraction Sums of Squared Loadings		
	Total	% of Variance	Cumulative %	Total	% of Variance	Cumulative %
1	**6.096**	32.082	32.082	6.096	**32.082**	32.082
2	**1.974**	10.388	42.471	1.974	**10.388**	42.471
3	**1.716**	9.033	51.504	1.716	**9.033**	51.504
4	**1.427**	7.512	59.016	1.427	**7.512**	**59.016**
5	0.960	5.054	64.069			
6	0.920	4.844	68.913			

Extraction Method: Principal Component Analysis. Values in bold represent the four components retained and their variance.

**Table 9:** Pattern Matrix.

	Component			
	1	2	3	4
Unskilled technical support	0.832			
Unavailability of specialist	0.829			
Unavailability of training capabilities	0.650			
Lack of adequate skills	0.624			
Lack of client insistence	0.599			
Lack of interest	0.540			
Prefer traditional method	0.403			
Technologies are too expensive		0.746		
Fear of change		0.644		
Lack of innovation		0.522		
Insufficient electricity			0.452	
Unavailability of financial resources			0.807	
Unavailability of funds from client			0.703	
Lack of cost to adopt			0.601	
Size of project			0.600	
Lack of education				0.795
Unalignment of labour supply				0.731
Fear of job losses				0.656
Lack of access to the wireless broadband				0.612

## Data Availability

Not applicable.
